# New insights into protein–DNA binding specificity from hydrogen bond based comparative study

**DOI:** 10.1093/nar/gkz963

**Published:** 2019-10-30

**Authors:** Maoxuan Lin, Jun-tao Guo

**Affiliations:** Department of Bioinformatics and Genomics, The University of North Carolina at Charlotte, Charlotte, NC 28223, USA

## Abstract

Knowledge of protein–DNA binding specificity has important implications in understanding DNA metabolism, transcriptional regulation and developing therapeutic drugs. Previous studies demonstrated hydrogen bonds between amino acid side chains and DNA bases play major roles in specific protein–DNA interactions. In this paper, we investigated the roles of individual DNA strands and protein secondary structure types in specific protein–DNA recognition based on side chain-base hydrogen bonds. By comparing the contribution of each DNA strand to the overall binding specificity between DNA-binding proteins with different degrees of binding specificity, we found that highly specific DNA-binding proteins show balanced hydrogen bonding with each of the two DNA strands while multi-specific DNA binding proteins are generally biased towards one strand. Protein-base pair hydrogen bonds, in which both bases of a base pair are involved in forming hydrogen bonds with amino acid side chains, are more prevalent in the highly specific protein–DNA complexes than those in the multi-specific group. Amino acids involved in side chain-base hydrogen bonds favor strand and coil secondary structure types in highly specific DNA-binding proteins while multi-specific DNA-binding proteins prefer helices.

## INTRODUCTION

Protein-DNA interactions play crucial roles in many cellular processes, such as transcription, DNA replication, DNA packaging and repair (1). Of particular interest is the specific recognition between proteins and DNA. Some DNA binding proteins are very specific, which include most type II restriction endonucleases, an important component of the restriction-modification (RM) systems in bacteria. These enzymes recognize and cleave foreign DNA at very specific target sequences while the target sites of the host DNA are protected from cleavage due to methylation (2). For example, EcoRI and BamHI, two widely used type II restriction endonucleases in molecular cloning, specifically recognize and cut the sequences GAATTC and GGATCC respectively. At the other end of DNA binding specificity spectrum, some DNA binding proteins, such as histone proteins and DNA polymerases, bind DNA non-specifically as they do not discriminate DNA sequences for binding. Transcription factors, a special group of DNA binding proteins, bind to specific and conserved DNA sequences while allowing variations at certain positions (3). It has been demonstrated that aberrant mutations or genetic variations can alter the binding specificity and thus affect the gene expression, leading to various types of diseases (4,5). Therefore, deciphering the protein–DNA recognition codes can not only help us better understand the mechanisms of these specific binding events, but also help explain diseases caused by mutations that affect protein–DNA binding specificity and design therapeutic drugs.

Over the last several decades, with the increasing number of high-resolution structures of protein–DNA complexes in Protein Data Bank (PDB) (6) and the advancement of technologies for exploring DNA binding motifs, such as ChIP-seq, protein-binding microarrays (PBMs) (7), systematic evolution of ligands by exponential enrichment combined with massively parallel sequencing (SELEX-seq) (8) and high-throughput SELEX (HT-SELEX) (9), our knowledge of protein–DNA binding specificity has been greatly expanded. DNA-binding proteins recognize their specific target sites with a combination of two readout mechanisms: base readout and shape readout (10,11). Base readout refers to the direct interaction between protein and DNA bases in major groove and minor groove, where the discrimination among bases can be achieved through shape fitting and electrostatic properties, including forming a number of key hydrogen bonds. While there is no simple one-to-one correspondence between amino acids and DNA bases, some particular amino acid-base pairings are enriched, such as arginine with guanine, and asparagine and glutamine with adenine (12–15). It has been shown that hydrogen bonds between amino acids and bases also provide complex interactions leading to specific recognition (16). Bidentate interactions, where two or more hydrogen bonds are formed between a residue and a base or a base pair, and complex interactions, where amino acids form hydrogen bonds with more than one base step, have been considered central to specific recognition of single base positions and short DNA sequences and are enriched in highly specific protein–DNA interactions (12,17,18). Recent studies also suggest that π-interactions between aromatic residues and DNA bases play important roles in specific protein–DNA recognition (17,19–22).

Shape readout refers to both global shape and local shape of target DNA sequences in protein–DNA recognition (10,23–28). DNA shape readout relies on both intrinsic and protein-induced DNA deformations in the core binding motifs as well as their flanking regions, especially the A- or T-rich stretch in the flanking regions (23,29,30). Recently, Rohs group investigated DNA shape changes due to CpG methylation and demonstrated these epigenetic effects on protein–DNA binding (31). They found that CpG methylation significantly alters local DNA shape, such as roll and propeller twist, and the degree of alterations is affected by the local sequence context. Another study on binding specificity of human transcription factors (TFs) using HT-SELEX and ChIP-seq revealed that homodimer orientation and spacing play a larger role in specific protein–DNA binding than previously thought (30). Based on these knowledge of protein–DNA binding specificity, various models have been developed for binding site prediction (20,24,30,32–35). While the performances of these models vary, adding shape features improves prediction accuracy over the sequence-only models.

Several recent studies have also investigated the roles of non-Watson-Crick (WC) base pairs, including Hoogsteen (HG) base pairs and mismatched (MM) base pairs, in protein–DNA recognition (36–38) (and Preprint at https://www.biorxiv.org/content/10.1101/705558v1). The tumor suppressor p53 recognizes diverse DNA response elements (REs) consisting of two continuous or interrupted decameric half-sites. Kitayner *et al*. found that the central A/T doublets of the conserved CATG motifs exhibited non-canonical HG base-pair geometry (37). This geometry affects the local shape and electrostatic potential of the B-DNA helix and hence the p53-DNA interface, leading to enhanced protein–DNA interactions. The HG geometry of the A/T doublets was also observed by Vainer *et al.* in crystal structure of Lys120-acetylated P53 DNA-binding domain in complex with consensus RE containing CATG motifs (38). Lys120 acetylation increases the flexibility of loop L1, which is known to increase the DNA-binding specificity of p53, and thus enables the formation of sequence-dependent DNA-binding models. To directly compare the effects of HG and WC base pairs on binding characteristics, Golovenko *et al*. studied p53-DNA crystal structures with designed REs having modified base pairs in either WC or HG form (36). They found that complexes with REs containing CATG motifs at the center of their half-sites favor the unique HG-induced shape and these complexes are more stable, resulting in enhanced interactions with p53. A very recent study reported the effect of DNA mismatches on DNA binding. The authors found while most MM base pairs within TF binding sites decreased or had no effect on binding affinity, a few MM base pairs increased binding affinity via inducing distortions similar to those induced by TF binding, pre-paying some of the energetic cost associated with DNA distortions contributing to recognition (Preprint at https://www.biorxiv.org/content/10.1101/705558v1). All these studies suggest non-Watson-Crick base pairs play larger roles in protein–DNA recognition than previously thought.

We recently carried out a comparative analysis of protein–DNA complex structures with different degrees of binding specificity (17). Our results revealed a clear trend of structural features among the three DNA-binding protein classes: highly specific (HS), multi-specific (MS), and non-specific (NS). DNA-binding proteins with higher binding specificity form more hydrogen bonds (including both simple and complex hydrogen bonds), have more major groove and base contacts, and the corresponding DNA shape harbors larger propeller and rise. In addition, we found that aspartate is enriched in highly specific DNA binding proteins and predominately binds to a cytosine through a single hydrogen bond or two consecutive cytosines through complex hydrogen bonds (17). Protein flexibility is another key factor in specific protein–DNA recognition (39–43). Highly specific and multi-specific DNA-binding domains tend to have larger conformational changes upon DNA binding and larger degree of flexibility in unbound states (17). Based on these observations, we developed a machine learning-based SVM (Support Vector Machine) model for TF (transcription factor)–DNA complex model assessment (44). The SVM model using structural features of specific protein–DNA interaction significantly improves prediction accuracy of TF–DNA complexes by successfully identifying cases without near-native structural models (44).

Current models for protein–DNA binding specificity primarily focus on interactions between protein and double-stranded DNA (dsDNA). Studies have shown that the double-stranded form of some DNA sequences and their corresponding single strands can serve as binding sites for different DNA-binding proteins (45–48). For example, the double-stranded form of a 30-bp asymmetric polypurine–polypyrimidine tract serves as a binding site for a transcription enhancer factor-1-related protein, while each single strand binds to two distinct protein factors in regulating the transcriptional activity of the mouse vascular smooth muscle alpha-actin gene in fibroblasts and myoblasts (45,48). Moreover, it has been reported that several sequence-specific DNA-binding transcription factors bind either the sense or antisense strands of some *cis*-regulatory elements with enhanced specificity (46,47). All these findings indicate that two DNA strands may play different roles in specific protein–DNA binding/recognition and the conservation at various binding positions.

We present here an investigation of protein–DNA binding specificity at DNA strand level with a particular focus on side chain-base hydrogen bonds since it has been demonstrated that side chain-base hydrogen bonds are critical to protein–DNA binding specificity (10,12,14,18). We first performed a comparative analysis at the strand level among DNA-binding proteins with different degrees of binding specificity, HS, MS and NS groups, to explore the contribution of each DNA strand to the overall protein–DNA binding specificity. Our hypothesis is that high binding specificity requires contributions from both DNA strands and thus the bases involved are highly conserved and more sensitive to mutations. In addition, we compared the secondary structure types of residues involved in side chain-base hydrogen bonds in different types of DNA-binding proteins and found distinct patterns. To our knowledge, this is the first large-scale comparative study of protein–DNA binding specificity at the DNA strand level and the role of secondary structure types in specific protein–DNA recognition.

## MATERIALS AND METHODS

### Datasets

The three groups of dsDNA-binding proteins with different degrees of binding specificity, HS, MS and NS, were compiled based on our previous study (17). Briefly, X-ray crystal structures of protein-dsDNA complexes with resolution ≤3 Å and *R*-factor ≤0.3 were selected from PDB. PDA (for protein–DNA complex structure Analyzer) was applied to reconstruct the complete DNA double helix structure via symmetry operations including rotation and translation for complexes with coordinates of only one strand of a double-stranded DNA (49). These complex structures were then annotated as HS, MS or NS DNA-binding domains based on their binding specificity and function of their DNA-binding domains. Complexes in each group were clustered using CD-HIT with a sequence identity cutoff of 30% (50). One representative from each cluster was selected to generate the non-redundant dataset (17). Since the original dataset contains a relatively small number of HS complexes, we expanded the HS dataset by adding four new non-redundant HS protein–DNA complex structures deposited in PDB since our last compilation (Supplementary Table S1). In addition, three DNA-binding domains were updated by either excluding the dimerization domains from the original annotations or by a new PDB ID. More specifically, domain 2e52D01 was changed from 2e52:D to 2e52:D (3–226) and domain 3lsrA01 was changed from 3lsr:A to 3lsr:A (4–53) (Supplementary Table S1). 3qws has been superseded by 6on0 in PDB on 15 May 2019. The final domain-based non-redundant dataset includes 32 HS, 115 MS and 52 NS protein–dsDNA complexes (17). For comparison purposes, in this study we also generated a corresponding chain-based dataset with 29 HS, 107 MS and 38 NS protein–dsDNA complexes (Supplementary Table S2).

### Hydrogen bonds and hydrogen bond energy

**Figure 1.**
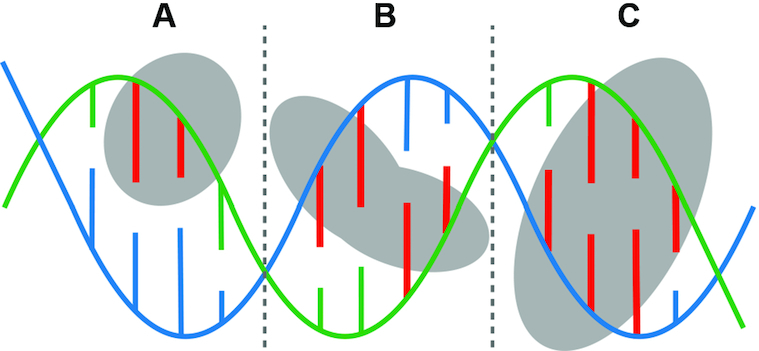
Schematic illustration of different types of side chain-base hydrogen bonds between two DNA strands (green and blue respectively) and a protein. The bases that form hydrogen bonds with protein side chain are colored red. (**A**) Hydrogen bonds between residue side chains and bases from only one DNA strand (green, the dominant strand); (**B**) equal number of bases that form hydrogen bonds with residue side chains from both DNA strands, also referred as a 50/50 case; (**C**) another 50/50 case with two base pair side chain-base hydrogen bonds.

To assess the contribution of each strand of the DNA double helix to binding specificity, we calculated the number of hydrogen bonds between residue side chains in DNA-binding proteins and DNA bases using HBPLUS (51) and FIRST (Floppy Inclusion and Rigid Substructure Topography) (52) with default parameters. To annotate the hydrogen bonds between protein and DNA with FIRST, we employed an energy cutoff of –0.6 kcal/mol as suggested by the author of FIRST (52). Percent contribution of each of the two DNA strands in a complex is calculated and the DNA strand with more hydrogen bonds is designated as the dominant strand. For example, the green strand in Figure 1A is the dominant strand. If both strands in complexes have equal number of bases forming side chain-base hydrogen bonds, either strand can be the dominant strand and these complexes are referred as 50/50 cases (Figure 1B and C). In some cases, both bases of a base pair are involved in forming side chain-base hydrogen bonds with the protein, and these hydrogen bonds are referred as base pair side chain-base hydrogen bonds (Figure 1C).

### Secondary structure types of DNA interacting residues

An amino acid is defined as a DNA base-contacting residue if it has at least one heavy atom of its side chain within 4.5 Å of any heavy atom of a DNA base. DSSP program was employed to assign three general secondary structure types: helix, strand and coil following the widely used convention: H (α-helix), G (3_10_-helix) and I (π-helix) states as helix type; E (extended strand) and B (residue in isolated β-bridge) states as strand type and all the other states from DSSP are considered as coil types (53–56).

### Statistical analysis

Shapiro–Wilk test was performed to test the normality of the data. If the data is normally distributed, a parametric Student's *t*-test was carried out. Otherwise, a non-parametric Wilcoxon rank-sum test was applied.

## RESULTS

### Comparison of hydrogen bonds between each strand of DNA and DNA-binding domains

It has been demonstrated that hydrogen bonds between amino acid side chains and DNA bases play major roles in specific protein–DNA interactions (10,12,14,18). It is not surprising that majority of the complexes in the non-specific (NS) DNA-binding group (34 out of 52 complexes) do not have any side chain-base hydrogen bonds and only five complexes have such hydrogen bonds between residues and bases in the major groove. Therefore, we focus on comparing the side chain-base hydrogen bonds between two groups of specific DNA-binding proteins with different degrees of binding specificity: HS and MS.

**Figure 2.**
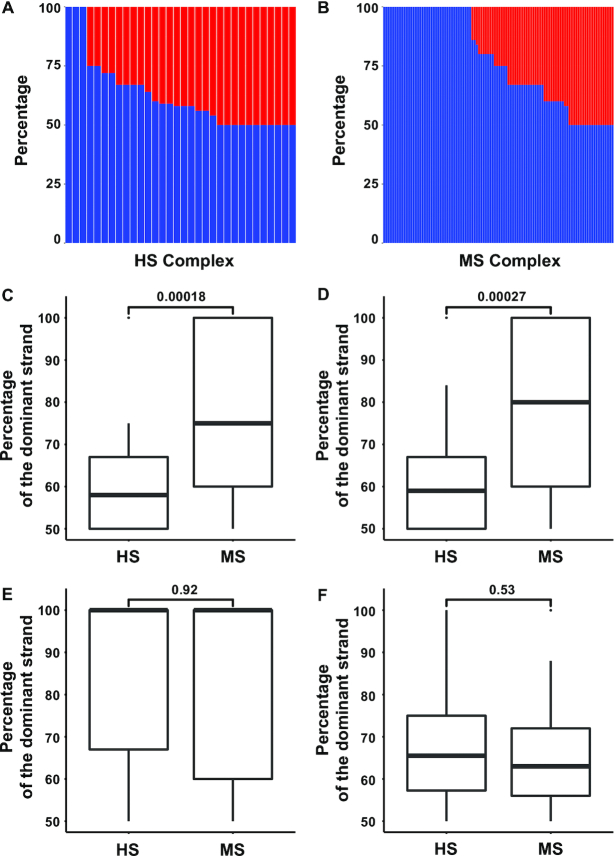
Comparison of the number of side chain-base hydrogen bonds of each strand of DNA annotated by HBPLUS between the HS and MS DNA-binding proteins. (**A**) Percentage contribution of two DNA strands in HS complexes; (**B**) percentage contribution of two DNA strands in MS complexes. The dominant strands (blue) are shown at the bottom in a descending order. Boxplots and statistical analyses for: (**C**) both major and minor grooves, (**D**) major groove only, (**E**) minor groove only and (**F**) non-side chain-base hydrogen bonds in both major and minor grooves. *P*-values are displayed on top of the boxplots.

Percent contributions of single DNA strands in each complex from HBPLUS are shown in Figure 2A and B, with the dominant strands shown at the bottom in a descending order. The two DNA strands of the complexes in the HS group tend to have equal or approximately equal contributions to the overall abundance of side chain-base hydrogen bonds. About 34% (11 of 32) of the HS cases have equal number of side chain-base hydrogen bonds from two strands of the DNA double helix and ∼91% (29 of 32) of the complexes have no more than 75% of the total contribution from the dominant DNA strand (Figure 2A). The MS group, on the other hand, only has ∼20% (20 of the total 102 complexes that have at least one side chain-base hydrogen bond) of the cases with equal contributions from the two DNA strands and ∼52% (53 of 102) of the complexes have no more than 75% of the total contribution from the dominant DNA strand (Figure 2B). Moreover, about 38% (39 of 102) of cases in the MS group only have side chain-base hydrogen bonds from one strand and zero from the other strand while less than 10% (3 of 32) of such cases are found in the HS group (Figure 2A, B).

Statistical analysis shows that the distributions of side chain-base hydrogen bonds between the HS and MS groups are significantly different for a combination of both major and minor grooves (Figure 2C) or for the major groove only (Figure 2D). The side chain-base hydrogen bonds in the minor groove are quite sparse and there are no apparent differences between HS and MS groups as they both skew towards one strand (Figure 2E). As a control, we compared distributions in terms of non-side chain-base hydrogen bonds from each strand, which are considered to contribute mainly to protein–DNA binding affinity but not much to specificity. Unlike the more specific side chain-base hydrogen bonds, there are no significant differences between the HS and MS groups, suggesting approximately equal contribution from each strand for hydrogen bonds between protein and DNA backbones in both HS and MS groups (Figure 2F). To make sure that these observations are robust and not biased results from HBPLUS, we applied a different hydrogen bond identification program, FIRST, using one suggested energy cutoff of –0.6 kcal/mol to determine the number of hydrogen bonds (52). Even though the total number of hydrogen bonds is slightly different from those annotated with HBPLUS due to different hydrogen bond identification algorithms, the results are nevertheless consistent with those from HBPLUS, which is two strands tend to contribute equally to the protein–DNA binding in terms of side chain-base hydrogen bonds in highly specific protein–DNA binding complexes, but the contribution skews towards one strand in the MS group (Supplementary Figure S1).

In addition to comparison of number of hydrogen bonds, we also carried out comparisons of hydrogen bond raw energy between two DNA strands since a hydrogen bond is identified as long as the hydrogen bond energy between two potential hydrogen bond forming atoms is below a cutoff value. The comparison of hydrogen bond energy (below cutoff –0.6 kcal/mol) from FIRST is shown in Supplementary Figure S2. Similar patterns to the number of hydrogen bonds were found between the HS and MS groups.

### Chain-based versus domain-based analyses

The above analyses were carried out between DNA-binding domains and DNA double helices. While some protein–DNA complexes only contain DNA-binding domains, other complexes consist of full-chain DNA-binding proteins, which may include signal-sensing or trans-activating domains besides DNA binding domains. These non-DNA-binding domains sometimes provide extra contacts between protein and DNA and contribute to protein–DNA binding affinity and/or binding specificity. It is interesting to see if there are any differences between domain-based and chain-based analyses with respect to the number of side chain-base hydrogen bonds from each DNA strand. While the numbers of hydrogen bonds and hydrogen bond energy are larger in the chain-based comparison, which is expected since some protein chains have two or more DNA binding domains, similar patterns of differences to the domain-based analyses are found between the HS and MS groups (Supplementary Figure S3). This is also in agreement with the findings reported by Jolma *et al.* that full-length transcription factors and isolated DNA-binding domains bind similar sequences and thus analysis of DNA-binding domains is sufficient to determine the protein–DNA binding specificity (30).

### DNA bases involved in hydrogen bonding with protein side chains from each DNA strand

**Figure 3.**
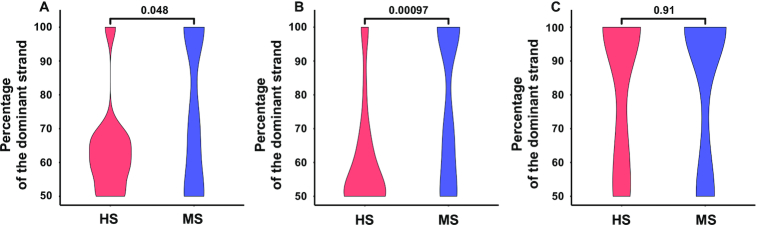
Comparison of the number of DNA bases involved in hydrogen bonding with side chains from HBPLUS for: (**A**) both major and minor grooves, (**B**) major groove only and (**C**) minor groove only, between HS and MS DNA-binding proteins.

Since some hydrogen bonds between DNA bases and protein side chains are bidentate and complex interactions, meaning one base can form two hydrogen bonds with one or more residues (12), we next compared the number of DNA bases that are involved in hydrogen bonding with amino acid side chains in DNA-binding domains between two DNA strands. The percentage of bases involved in side chain-base hydrogen bonding from the dominant strands is close to 50% in the HS group while it is larger in the MS group when base contacts in both major and minor grooves are considered (Figure 3A) or only base contacts in the major groove are considered (Figure 3B). Similar results are observed with FIRST (Supplementary Figure S4A and B). The *P*-value in Figure 3A that compares the number of bases involved in side chain-base hydrogen bonding in both major and minor grooves with HBPLUS is slightly higher (but still <0.05). A closer examination of the data revealed that HBPLUS identifies more complexes and more bases that form hydrogen bonds with side chains in the minor groove than those from FIRST, resulting in a larger percentage of complexes in the MS group with smaller percentage contributions from the dominant strands (data not shown). No apparent differences were found in the minor groove (Figure 3C and Supplementary Figure S4C).

### Side chain-base hydrogen bonding base pairs

**Figure 4.**
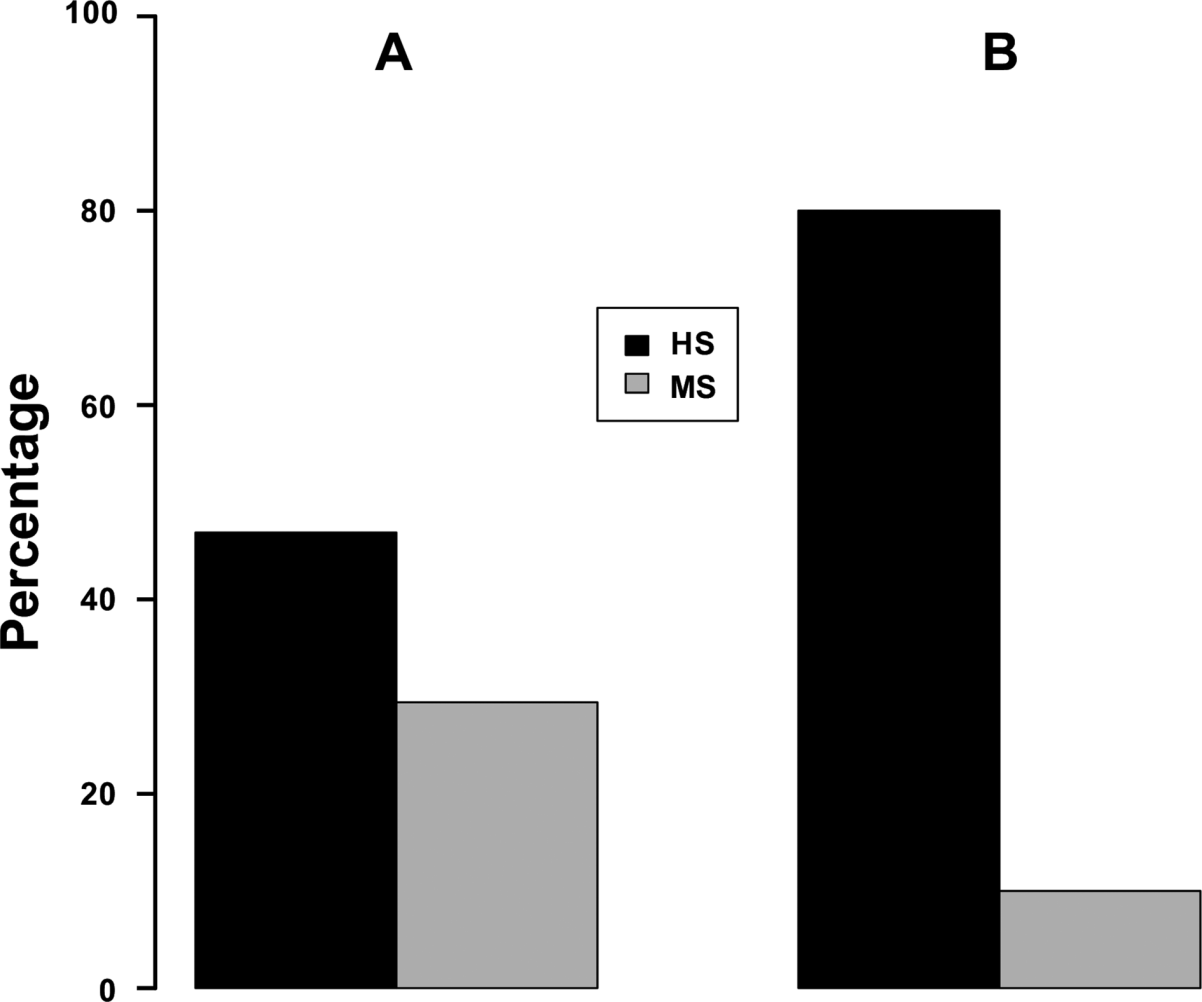
Comparison of the number of 50/50 cases (**A**) and the number of cases with base pairs involved in hydrogen bonding with residue side chains from these 50/50 cases (**B**) between the HS group and MS group with HBPLUS. (**A**) The percentage was calculated by dividing the number of 50/50 cases in each group over the total number of complexes forming side chain-base hydrogen bonds in that group; (**B**) the proportion of these 50/50 cases that have base pairs involved in side chain-base hydrogen bonding.

**Figure 5.**
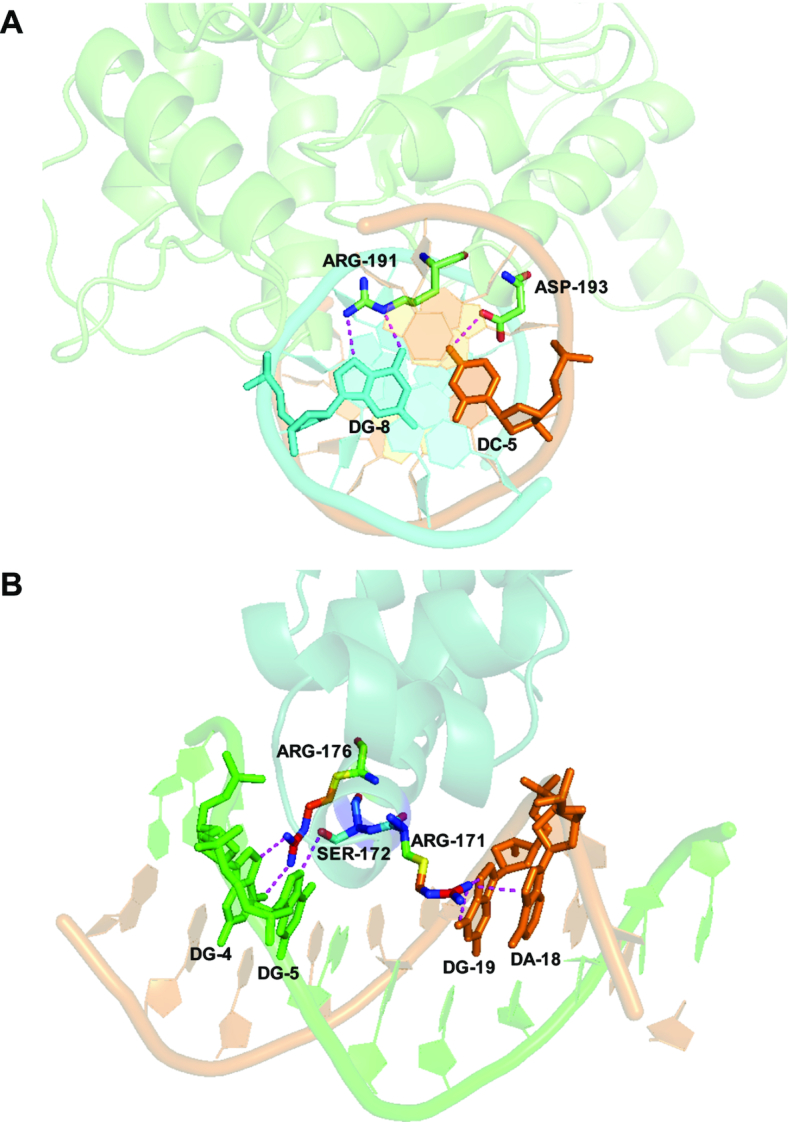
Examples of DNA-binding proteins bound to paired bases and unpaired bases. (**A**) Highly specific DNA-binding protein NgoMIV bound to paired bases (PDBID: 4ABT; protein chain: A; DNA chains: E and H). Only one out of three continuous base pairs involved hydrogen bonding is highlighted. Base pairs DC-9 (chain E) and DG-4 (chain H), DG-7 (chain E) and DC-6 (chain H) are also involved in side chain-base hydrogen bonds. (**B**) Multi-specific DNA-binding protein sigma(E)4 bound to equal number but unpaired bases with two strands (PDBID: 2H27; protein chain: A; DNA chains: B and C).

**Figure 6.**
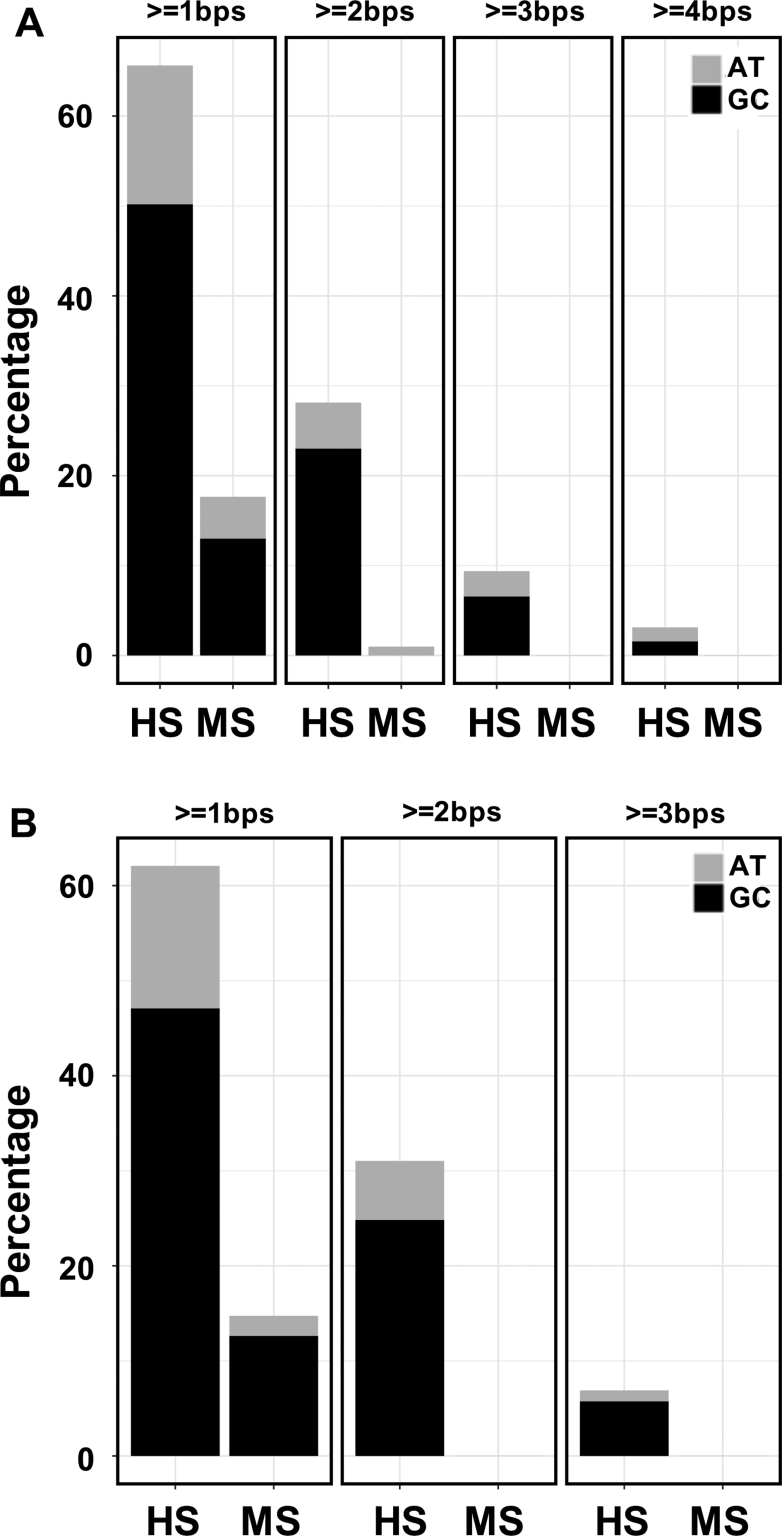
Comparison of base pairs that are involved in side chain-base hydrogen-bonding between HS and MS groups with HBPLUS in (**A**) both major and minor grooves and (**B**) major groove only.

Not only does the HS group have much larger percentage of complexes (15/32 ≈ 47%) that have equal number of bases forming side chain-base hydrogen bonds in the major groove from two DNA strands (50/50 cases) than the MS group (30/102 ≈ 29%) (Figure 4A), the majority of these 50/50 cases in the HS group have base pair side chain-base hydrogen bonds (12/15 = 80%), while only 3 out of 30 (10%) cases in the MS group have base pairs forming hydrogen bonds with protein side chains (Figure 4B and Supplementary Figure S5). For instance, while both restriction endonuclease NgoMIV (PDBID: 4ABT) and transcription factor *Escherichia coli* sigma(E)4 (PDBID: 2H27) form side chain-base hydrogen bonds with equal number of bases from two DNA strands in the major groove, the highly specific DNA binding protein NgoMIV has three continuous base pairs involved in forming hydrogen bonds (Figure 5A and Supplementary Figure S5A) but the multi-specific sigma(E)4 forms such hydrogen bonds with unpaired bases (Figure 5B and Supplementary Figure S5B). The 50/50 cases from FIRST annotations show similar results with 8/13 ≈ 62% in the HS group and 4/24 ≈ 17% in the MS group (Supplementary Figure S6). The total amounts of base pairs involved in hydrogen bonding with residues are shown in Figure 6 (HBPLUS) and Supplementary Figure S7 (FIRST). The HS group has much larger percentage of complexes that have at least one base pair, two or more base pairs that are involved in side chain-base hydrogen bonding than the MS group. GC base pairs are more prevalent than AT base pairs in both HS and MS groups.

### Secondary structure types of DNA interacting residues

**Figure 7.**
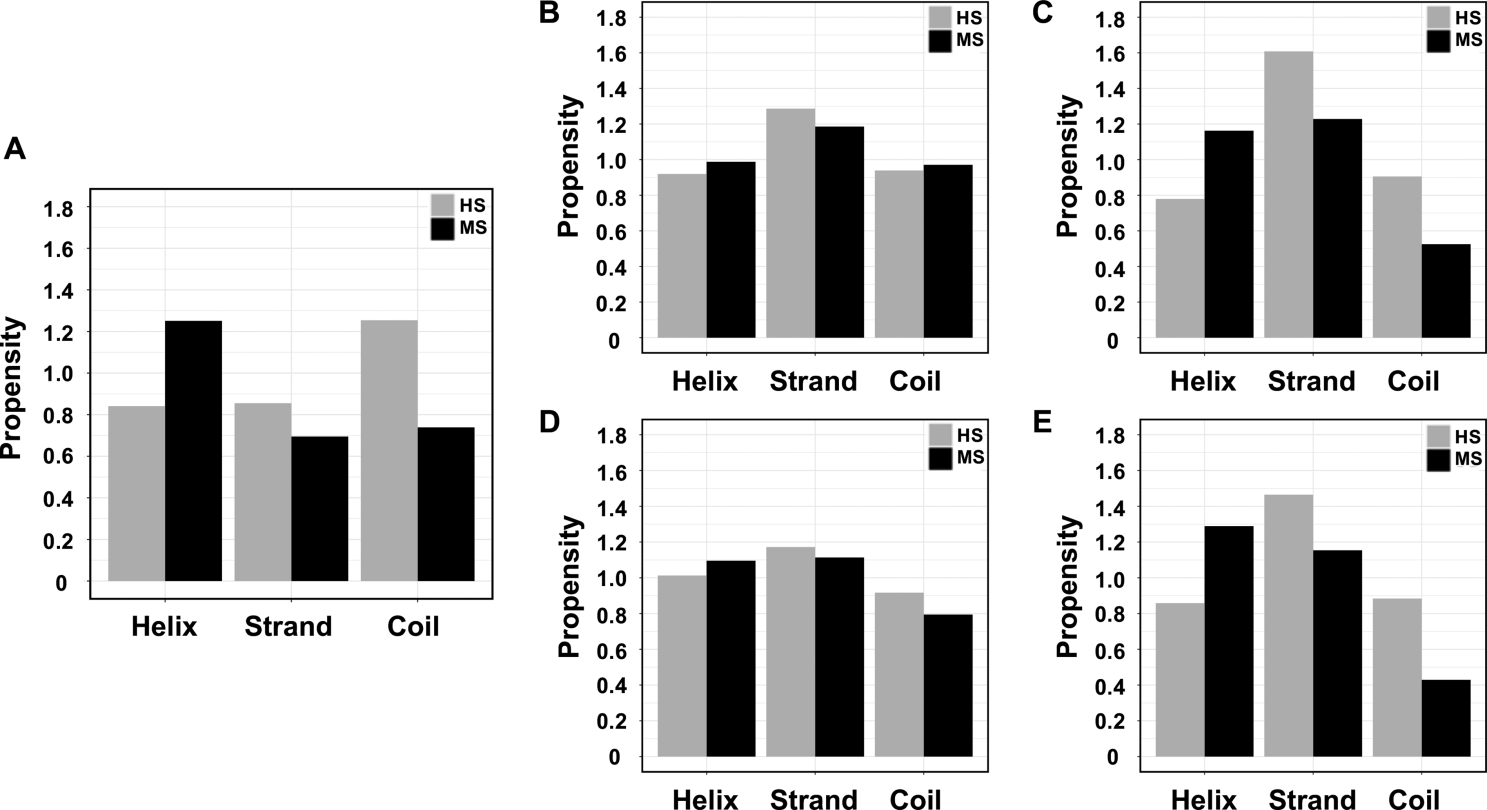
Propensities of secondary structure types in the HS and MS groups. (**A**) Propensities of secondary structure types of DNA base-contacting residues, the background relative frequencies of secondary structure types are calculated using all residues in the DNA-binding domains in each group. Propensities of secondary structure types of residues involved in side chain-base hydrogen bonds with HBPLUS for both major and minor grooves (**B, D**) and for major groove only (**C, E**). Propensities are calculated using either the relative frequencies of secondary structure types of base-contacting residues (**B, C**) or all DNA hydrogen-bonding residues (D, E).

DNA-binding proteins recognize their target sites with a number of common binding motifs, such as helix-turn-helix, ββα zinc finger and zipper-type motifs (1). The secondary structure types of amino acids involved in specific protein–DNA binding, however, have not been investigated extensively. We first compared the propensities of the secondary structure types of amino acids in DNA-binding domains that are in contact with DNA bases, calculated against the relative frequencies of secondary structure types of residues in respective group of DNA-binding domains. The DNA base-contacting residues in the HS group are enriched in coil conformations while helical secondary structure types are preferred in the MS group (Figure 7A). For residues that form hydrogen bonds between their side chains and DNA bases, we used two different background distributions to calculate the propensities: one is the secondary structure type distribution of all base-contacting residues (Figure 7B and C) and the other is the secondary structure type distribution of all residues that form hydrogen bonds with DNA including bases and backbone atoms (Figure 7D and E).

When residues involved in side chain-base hydrogen bonds in the major and minor grooves are combined, DNA-binding proteins in both the HS and MS groups prefer strand types and there are no major differences between the HS and MS groups no matter which background distribution is used (Figure 7B and D). However, when only such contacts in the major groove are considered, there is a distinct pattern. The strand type is highly enriched in the HS group, while proteins in the MS group favor both strand and helical types but are depleted in coil conformations when compared to DNA-binding domains in the HS group (Figure 7C and E). For example, residues involved in side chain-base hydrogen bonds in restriction endonuclease BstYI, a highly specific DNA-binding protein, reside in strand and coil secondary structure types (Supplementary Figure S8A) while in hepatocyte nuclear factor 1-alpha (HNF-1alpha) residues in helical conformation are involved in hydrogen bonding with bases (Supplementary Figure S8B). The above results suggest a role of flexibility in conferring different degrees of binding specificity (See detailed discussions in the next section). This observation is consistent between HBPLUS and FIRST results (Supplementary Figure S9). Further investigation revealed that residues in the MS group that are involved in side chain-base hydrogen bonds have ∼70% coils in the minor groove, which may explain the differences of propensities between the major+minor grooves (Figure 7B and D) and major groove alone (Figure 7C and E).

## DISCUSSION

Understanding the mechanisms of protein–DNA binding specificity is of paramount importance in deciphering gene regulation networks and designing therapeutic drugs. It has been demonstrated that hydrogen bonds between amino acid side chains and DNA bases play major roles in specific protein–DNA recognition (10,12,14,18). As such, to further understand structural features in protein–DNA binding specificity, we performed a comparative analysis based on side chain-base hydrogen bonds. We first investigated protein–DNA binding specificity at DNA strand level, which has not been explored before. The amounts of side chain-base hydrogen bonds between each DNA strand and DNA-binding domains of two groups of DNA-binding proteins, HS and MS, were compared (17). Since there are a number of different algorithms for calculating hydrogen bond energy and typically a default energy cutoff is applied for determining the existence of hydrogen bonds, we applied two widely used hydrogen bond annotation programs HBPLUS and FIRST to ensure our results are robust and the conclusions are independent of hydrogen bond identification programs. Results show that DNA-binding domains with high binding specificity have approximately equal contributions of side chain-base hydrogen bonds from two DNA strands, while a larger percentage of protein–DNA complexes form side chain-base hydrogen bonds with only one DNA strand in the MS group (Figure 2, Supplementary Figure S1). Not only are these findings in agreement between HBPLUS and FIRST, they are also consistent between domain-based and chain-based analyses (Supplementary Figure S3).

We also found that highly specific protein–DNA complexes have more base pairs involved in hydrogen bonding with protein side chains than those with lower binding specificity in the MS group (Figure 6 and Supplementary Figure S7). These observations, approximately equal distributions from two DNA strands and larger number of base pairs involved in side chain-base hydrogen bonding in the high binding specificity group, help explain why the bases in the high binding specificity group are highly conserved and are very sensitive to mutations. DNA-binding proteins in the HS group are mainly Type II restriction endonucleases. These endonucleases recognize short palindromic sequences of 4–8 bps specifically as homodimers and cleave DNA double helices (57). This process relies on the concerted recognition of two DNA strands and the communication of this recognition information between two subunits, suggesting this recognition process coordinates efforts from specific interactions between protein and both DNA strands. Transcription factors in the MS group, on the other hand, regulate gene expression by binding to target sequences, called transcription factor binding sites (TFBS) (58). While the binding between transcription factors and their corresponding binding sites is specific and certain positions are highly conserved, transcription factors generally allow variability at some other base positions. In addition, it has been shown that some transcription factors can bind to two different binding motifs, called primary and secondary binding motifs (59). If one strand is the primary one for a DNA-binding protein, a base mutation would have less effect than the case that both bases of a base pair get involved in specific interaction. Hydrogen-bonding donor and acceptor patterns in the major groove are unique to specific base pairs, therefore it is impossible to maintain the original hydrogen-bonding patterns if a base of a base pair is mutated when this particular base pair is involved in specific hydrogen bonding, making it more sensitive to mutations and thus more conserved.

Majority of the base pairs involved in hydrogen bonding in both HS and MS groups are GC pairs (Figure 6 and Supplementary Figure S7). Nadassy *et al.* analyzed 65 X-ray structures of protein-dsDNA complexes and observed that GC pairs make three times as many hydrogen bonds as AT pairs in the major groove (60). However, in their study, the occurrence of base pairs was counted in a different way. As long as one base of a pair is involved, it is considered a pair participation. Nikolajewa *et al.* found a significant GC contact in type II restriction enzyme binding sites (61). These results suggest that GC pairs play critical roles in specific protein–DNA binding. These observations are not surprising since guanine has a strong electronegative character in the major groove and is compatible to the guanidinium group of arginine. In addition, guanine contributes an extra hydrogen bond donor of N2 in the minor groove. Studies have shown that the addition, removal, substitution and relocation of the exocyclic 2-amino group of guanine in the minor groove affect DNA cleavage by DNA-binding proteins, DNA binding with small molecules and antibiotics (62–65). For instance, by examining base substitutions that affect the presence and location of the 2-amino group of guanine in *tyr*T(A93) DNA, Bailly *et al.* found these alterations affect both the flexibility of *tyr*T(A93) DNA and its affinity for its binding protein, the *Escherichia coli* Factor for Inversion Stimulation (FIS) (65).

Statistical analyses show significant differences in the major groove but not in the minor groove between HS and MS groups. This is consistent with the base readout mechanism. In the major groove, every base pair has a unique hydrogen bond acceptor and donor pattern that can be distinguished from other base pairs. In the minor groove, however, the degeneracy of the pattern of hydrogen bond acceptors and donors cannot distinguish A/T from T/A or C/G from G/C. For non-specific DNA-binding proteins, we found more complexes have side chain-base hydrogen bonds in the minor groove than the major groove (data not shown). Although in general hydrogen bonds between proteins and bases in the minor groove play a less role than those in the major groove, in some cases, the minor groove hydrogen bonds are critical especially when the shape readout is considered. Rohs *et al.* demonstrated that arginine prefers to bind narrow minor grooves in AT-rich regions and the role of DNA shape in the protein–DNA recognition, which represents a novel DNA recognition mechanism in many DNA binding protein families (26). These minor-groove interactions may stabilize the deformed DNA structure and identify incorrectly incorporated non-Watson-Crick base pairs (66). It has also been reported that amino acid side chain-base hydrogen bonds in the minor groove are important in insertion and extension of base pairs in DNA replication (67–70).

DNA base-contacting residues in highly specific DNA-binding proteins are enriched in coils while multi-specific DNA-binding proteins prefer helices (Figure 7A). For residues forming hydrogen bonds with bases in the major groove, the propensity of coil conformations for HS proteins is about two times more than that for the MS proteins (Figure 7C and E, Supplementary Figure S9B and D). These results suggest that protein flexibility play important roles in protein–DNA recognition, as reported in previous studies (17,39–43). For instance, our previous study found that specific DNA-binding domains tend to have larger conformational changes upon DNA-binding and larger degree of flexibility in unbound states (17). It has been hypothesized that protein flexibility can help speed up DNA recognition (71,72). The higher flexibility of coils than helices should play important roles in locating DNA-binding proteins to their specific target sites. More importantly, flexibility can enhance the binding specificity via forming larger number of hydrogen bonds with DNA bases due to coil's fine-tuning capability. A recent comparative molecular dynamics simulations on wild-type and F10V mutant P22 Arc repressor in both free and complex conformations demonstrated the role of protein flexibility in protein–DNA binding specificity (42). The DNA-binding motif of wild-type Arc repressor is more flexible and this flexibility leads to more hydrogen bonds formed with DNA bases upon binding, which results in higher DNA-binding specificity (42). We also found that while residues involved in hydrogen bonding with DNA major grooves generally prefer strand secondary structure types (HS group shows slightly higher preference), MS group also favors helices (Figure 7C and E). Mutation tolerance study of different secondary structure elements of proteins shows that alpha helices are more robust to mutations than beta strands (73). The preference of strands of highly specific DNA-binding proteins makes them more sensitive to mutations from the perspective of protein conformations. These secondary structure type preferences and the fact that DNA bases are more conserved in highly specific DNA-binding proteins, indicate that the conservation of highly specific DNA-binding proteins requires both conserved protein secondary structures and DNA bases.

While our analyses are based on complexes with targeted DNA bases forming canonical Watson-Crick base pairing geometry, the method can be generalized for studying structures with non-Watson-Crick base pairs, including HG and MM base pairs when large datasets of such cases become available. In addition to DNA shape, the effect of DNA mismatches on protein–DNA binding specificity can be investigated in terms of hydrogen bonds (https://www.biorxiv.org/content/10.1101/705558v1). It would be interesting to see how the mutated bases of those mismatched base pairs from different strands affect the protein–DNA binding affinity and/or specificity by altering the hydrogen bonding patterns or other types of interactions. Anti-syn transitions of DNA base conformation have been widely observed when base pairing changes from WC geometry to HG and MM base pairing (74–77). Future studies can reveal if the transitions are biased toward one strand or randomly distributed between two strands. Our results also offer possible clue to the increased mutation rates around transcription factor binding sites (TFBS) (78,79). The increased levels of mutations around TFBS have been attributed to the barrier created by DNA-binding proteins to the displacements of DNA synthesized by error-prone polymerase-α (78), and a decrease of nucleotide excision repair (NER) activity caused by interference of DNA-binding proteins with the NER machinery (79).

Our study, for the first time to our knowledge, reports that high protein–DNA binding specificity may require approximately equal contributions from two DNA strands. Investigation of secondary structure types of DNA interacting residues suggests that both secondary structure types and protein flexibility play important roles in specific protein–DNA recognition. Our results not only provide new insights into protein–DNA binding specificity, but also have great potential in further exploration of novel mechanisms of protein–DNA interactions in complexes containing non-Watson-Crick base pairs.

## Supplementary Material

gkz963_Supplemental_File
