# Supplementary material for: New insights into protein–DNA binding specificity from hydrogen bond based comparative study
Source: Nucleic Acids Res. 2019 Oct 30;47(21):11103–13. doi: 10.1093/nar/gkz963 (PMC6868434; doi:10.1093/nar/gkz963)

Table S1. Domain-based non-redundant DNA-binding domains in HS, MS and NS groups.

Dataset	Domain ID	Domain definition	Domain ID	Domain definition
HS	1az0B00	1az0:B	d1yfib_	1yfi:B
	1bhmA00	1bhm:A	2e52D01*	2e52:D (3-226)
	1d2iB00	1d2i:B	h3m7kA0	3m7k:A
	1dc1A01	1dc1:A (5-38,127-323)	h3oqgA0	3oqg:A
	1dc1A02	1dc1:A (39-126)	m2fl3A0	2fl3:A
	1eriA00	1eri:A	m2oaaA0	2oaa:A
	1iawA01	1iaw:A (10-176)	m3c25A0	3c25:A
	1iawA02	1iaw:A (177-309)	m3fc3B1	3fc3:B (2-107)
	1kc6B00	1kc6:B	m3goxB2	3gox:B (108-189)
	1pviA00	1pvi:A	m3imbD0	3imb:D
	1vrrA00	1vrr:A	m3ndhA0	3ndh:A
	1wteA01	1wte:A (1-87, 212-272)	m4rdmB0	4rdm:B
	1wteA02	1wte:A (88-211)	**m2vlaA0**	**2vla:A**
	3dvoD00	3dvo:D	**m4zsfA1**	**4zsf:A(70-272)**
	3hqfA00	3hqf:A	**m5dwaA0**	**5dwa:A**
	4abtA00	4abt:A	**m6ekoA0**	**6eko:A**
MS	1b3tA00	1b3t:A	1nkpD00	1nkp:D
	1bdtD00	1bdt:D	1owrP01	1owr:P (397-569)
	1bl0A01	1bl0:A (9-64)	1pnrA01	1pnr:A (3-59)
	1bl0A02	1bl0:A (65-124)	1qn3B01	1qn3:B (19-29, 116-197)
	1cf7A00	1cf7:A	1qn6A02	1qn6:A (30-115)
	1cmaA00	1cma:A	1qpiA01	1qpi:A (4-66)
	1ea4G00	1ea4:G	1r8dA00	1r8d:A
	1exjA02	1exj:A (3-75)	1rioH00	1rio:H
	1fzpB00	1fzp:B	1saxA01	1sax:A (9-72)
	1gd2E00	1gd2:E	1sknP00	1skn:P
	1gxpE00	1gxp:E	1t2kB01	1t2k:B (7-110)
	1h6fA00	1h6f:A	1xpxA00	1xpx:A
	1hjbB00	1hjb:B	1zreA02	1zre:A (138-207)
	1hjbC00	1hjb:C	1zs4A00	1zs4:A
	1ic8A01	1ic8:A (87-180)	2ac0C00	2ac0:C
	1ic8A02	1ic8:A (203-276)	2bopA00	2bop:A
	1jfiA00	1jfi:A	2e1cA01	2e1c:A (24-76)
	1jfiB00	1jfi:B	2h27A00	2h27:A
	1k78A01	1k78:A (19-84)	2h7hA00	2h7h:A
	1k78B00	1k78:B	2i9tB02	2i9t:B (546-650)
	1kb2A00	1kb2:A	2p5lC00	2p5l:C
	1le5F01	1le5:F (38-241)	2r5yB00	2r5y:B
	1lmb300	1lmb:3	2wt7A00	2wt7:A
	1lq1B00	1lq1:B	2yvhD00	2yvh:D
	1mdmA02	1mdm:A (85-139)	2zhgA00	2zhg:A
	1mhdA00	1mhd:A	3a01A00	3a01:A

MS	3coaC00	3coa:C	h3mlpE0	3mlp:E
	3dfxB00	3dfx:B	h3vebA0	3veb:A
	3dnvB00	3dnv:B	h3w3cA0	3w3c:A
	3g97A00	3g97:A	h3zplF0	3zpl:F
	3hddB00	3hdd:B	h4gclD0	4gcl:D
	3iagC01	3iag:C (53-200, 359-380)	h4h10A0	4h10:A
	3iagC02	3iag:C (201-358)	h4hf1A0	4hf1:A
	3iktA01	3ikt:A (0-73)	h4ihtC0	4iht:C
	3jtgA01	3jtg:A (273-357)	h4ix7A0	4ix7:A
	3jxdR00	3jxd:R	h4jl3A0	4jl3:A
	3o9xA02	3o9x:A (59-131)	m3fdqA0	3fdq:A
	3p57B01	3p57:B (13-91)	m3h0dB1	3h0d:B (3-75)
	3pvvB00	3pvv:B	m3n7qA0	3n7q:A
	6on0A00#	6on0:A	m3u3wA1	3u3w:A (3-58)
	3s8qA00	3s8q:A	m3w6vA0	3w6v:A
	3u2bC00	3u2b:C	m3zqlA0	3zql:A
	3zkcB00	3zkc:B	m4g92A0	4g92:A
	4fthA00	4fth:A	m4g92C0	4g92:C
	4g92B00	4g92:B	m4jcyB0	4jcy:B
	6croA00	6cro:A	m4knyA2	4kny:A (124-225)
	d1odha_	1odh:A	m4l62P1	4l62:P (7-49)
	d2iszd1	2isz:D (1-64)	m4ldxB2	4ldx:B (121-229)
	d2xsdc1	2xsd:C (247-319)	m4llnA0	4lln:A
	d2xsdc2	2xsd:C (343-397)	m4lmgD0	4lmg:D
	d3coqa1	3coq:A (8-48)	m4mteB1	4mte:B (3-72)
	d3e6cc1	3e6c:C (148-233)	m4nnuA1	4nnu:A (44-122)
	h2er8C0	2er8:C	m4nnuA3	4nnu:A (153-236)
	h2vy1A0	2vy1:A	m4on0B0	4on0:B
	h3a5tA0	3a5t:A	m4qtkA0	4qtk:A
	h3gnaA0	3gna:A	m4u0yB0	4u0y:B
	h3igmA0	3igm:A	m4ux5A0	4ux5:A
	3lsrA01*	3lsr:A (4-53)		
NS	1cezA01	1cez:A (8-325)	1ya6B01	1ya6:B (998-1176, 1387-1400)
	1f66C00	1f66:C	2bzfA00	2bzf:A
	1jeyA02	1jey:A (251-278, 342-439)	2dnjA00	2dnj:A
	1jeyA03	1jey:A (279-341)	2pi4A05	2pi4:A (554-784)
	1jeyB02	1jey:B (243-443)	2voaA00	2voa:A
	1rztA03	1rzt:A (386-508)	2wtfA04	2wtf:A (393-509)
	1rztI04	1rzt:I (509-575)	3aafA00	3aaf:A
	1skrA03	1skr:A (415-477, 590-704)	3av2A00	3av2:A
	1sxqA02	1sxq:A (167-332)	3cwsC02	3cws:C (113-230)
	1x9wA02	1x9w:A (233-414)	3gv5B04	3gv5:B (299-414)
	1xslA02	1xsl:A (332-385)	3l4jA01	3l4j:A (429-561, 609-691)

NS	3l4jA03	3l4j:A (692-860, 974-988)	h4o0iA2	4o0i:A (491-605)
	3l4jA04	3l4j:A (872-973)	h4o5eA3	4o5e:A (149-335)
	3n4mB00	3n4m:B	h4oinD0	4oin:D
	3uiqA02	3uiq:A (109-339)	m2o8bA3	2o8b:A (321-855)
	3uiqA06	3uiq:A (775-866)	m2o8bB1	2o8b:B (362-518)
	4eyhB01	4eyh:B (26-36, 99-221)	m2o8bB3	2o8b:B (728-1335)
	d3jxya_	3jxy:A	m3f2bA0	3f2b:A
	d4klua1	4klu:A (11-91)	m3l2pA1	3l2p:A (168-336)
	d9icka3	9ick:A (92-148)	m4c2uA4	4c2u:A (384-561)
	h1s9fA4	1s9f:A (244-341)	m4dl4A4	4dl4:A (313-432)
	h2wwyA0	2wwy:A	m4ir1F1	4ir1:F (0-10, 74-165)
	h3kxtA0	3kxt:A	m4ir1F4	4ir1:F (236-341)
	h3raxB3	3rax:B (1167-1233)	m4o3mA3	4o3m:A (1072-1194)
	h4eluA2	4elu:A (423-832)	m4plbB1	4plb:B (417-1033)
	h4g0vB0	4g0v:B	m4plbB2	4plb:B (1034-1376, 1461-1491)

* DNA-binding domain was updated by excluding dimerization domain.

# 6on0 superseded 3qws on 2019-05-15.

Bold: New HS DNA-binding domains. Their names and recognition sequences are:

m2vlaA0	BpuJI(CCCGT)
m4zsfA1	BsaWI(W^CCGGW)
m5dwaA0	AgeI(A^CCGGT)
m6ekoA0	PfoI(T^CCNGGA)

Table S2. Chain-based non-redundant protein-dsDNA complexes in HS, MS and NS groups.

Dataset	PDBID (Protein-chain_DNA-chains)
HS	1AZ0(B_CD), 1BHM(A_CD), 1D2I(B_CD), 1DC1(A_CW),<br>1ERI(A_BC), 1IAW(A_EF), 1IAW(A_CD), 1KC6(B_EF),<br>1PVI(A_CD), 1VRR(A_CD), 1WTE(A_XY), 3DVO(D_GH),<br>3HQF(A_BC), 4ABT(A_EH), 1YFI(B_EF), 2E52(D_FH),<br>3M7K(A_BC), 3OQG(A_CD), 2FL3(A_CD), 2OAA(A_CD),<br>3C25(A_CD), 3FC3(B_CD), 3IMB(D_KL), 3NDH(A_CD),<br>4RDM(B_EF), 2VLA(A_LM), 4ZSF(A_BD), 5DWA(A_CD),<br>6EKO(A_EF)
MS	1B3T(A_CD), 1BDT(D_EF), 1BL0(A_BC), 1CF7(A_CD),<br>1CMA(A_CD), 1EA4(G_WX), 1EXJ(A_BD), 1FZP(B_KW),<br>1GD2(E_AB), 1GXP(E_GH), 1H6F(A_CD), 1HJB(B_GH),<br>1HJB(C_GH), 1IC8(A_EF), 1JFI(A_DE), 1JFI(B_DE),<br>1K78(A_CD), 1K78(B_CD), 1KB2(A_CD), 1LE5(F_GH),<br>1LMB(3_12), 1LQ1(B_GH), 1MHD(A_CD), 1NKP(D_HJ),<br>1OWR(P_EF), 1PNR(A_BD), 1QN3(B_EF), 1R8D(A_CD),<br>1RIO(H_TU), 1SAX(A_CD), 1SKN(P_AB), 1T2K(B_EF),<br>1XPX(A_CD), 1ZRE(A_WX), 1ZS4(A_TU), 2AC0(C_GH),<br>2BOP(A_BC), 2E1C(A_BD), 2H27(A_BC), 2H7H(A_XY),<br>2P5L(C_AB), 2R5Y(B_CD), 2WT7(A_CD), 2YVH(D_EF),<br>2ZHG(A_BC), 3A01(A_CD), 3COA(C_AB), 3DFX(B_XY),<br>3G97(A_CD), 3HDD(B_CD), 3IAG(C_AB), 3IKT(A_CD),<br>3JTG(A_BC), 3JXD(R_AB), 3O9X(A_EF), 3P57(B_EF),<br>3PVV(B_EF), 6ON0(A_CN), 3S8Q(A_CD), 3U2B(C_AB),<br>3ZKC(B_CD), 4FTH(A_CD), 4G92(B_DE), 6CRO(A_RU),<br>1ODH(A_CD), 2ISZ(D_EF), 2XSD(C_AB), 3COQ(A_DE),<br>3E6C(C_AB), 2ER8(C_GH), 2VY1(A_CW), 3A5T(A_CD),<br>3IGM(A_CD), 3LSR(A_BD), 3MLP(E_GH), 3VEB(A_MN),<br>3W3C(A_BC), 3ZPL(F_GH), 4GCL(D_WZ), 4H10(A_CD),<br>4HF1(A_CD), 4IHT(C_GH), 4IX7(A_CD), 4JL3(A_EF),<br>3FDQ(A_CD), 3H0D(B_CD), 3N7Q(A_BC), 3U3W(A_YZ),<br>3W6V(A_BC), 3ZQL(A_EF), 4G92(A_DE), 4G92(C_DE),<br>4JCY(B_CD), 4KNY(A_YZ), 4L62(P_WX), 4LDX(A_CD),<br>4LLN(A_GH), 4LMG(D_GH), 4MTE(B_YZ), 4NNU(A_CD),<br>4ON0(B_EF), 4QTK(A_CD), 4U0Y(B_EF), 4UX5(A_CD),<br>1QPI(A_BM), 3DNV(B_ET), 3GNA(A_DE)
NS	1CEZ(A_NT), 1F66(C_IJ), 1JEY(A_CD), 1JEY(B_CD),<br>1RZT(A_BC), 1RZT(A_NO), 1SXQ(A_CE), 1X9W(A_CD),<br>1YA6(B_CD), 2BZF(A_BC), 2DNJ(A_BC), 2VOA(A_CD),<br>2WTF(A_OP), 3AAF(A_CD), 3AV2(A_IJ), 3CWS(C_GH),<br>3GV5(B_PT), 3L4J(A_BC), 3N4M(B_DE), 3UIQ(A_PT),<br>3JXY(A_BC), 4KLU(A_DT), 2WWY(A_PQ), 3KXT(A_BC),<br>3RAX(B_HJ), 4ELU(A_BC), 4G0V(B_DE), 4O0I(A_BC),<br>4OIN(D_GH), 2O8B(A_EF), 2O8B(B_EF), 3F2B(A_PT),<br>3L2P(A_BD), 4C2U(A_XY), 4DL4(A_PT), 4IR1(F_GH),<br>4O3M(A_PT), 4PLB(B_EF)

Figure S1. Comparison of the number of side chain-base hydrogen bonds annotated by FIRST (-0.6 kcal/mol cutoff) of each strand of DNA between the HS and MS DNA-binding proteins. (A) Percentage contribution of two DNA strands in HS complexes; (B) Percentage contribution of two DNA strands in MS complexes. The dominant strands (blue) are shown at the bottom in a descending order. Boxplots and statistical analyses for: (C) both major and minor grooves, (D) major groove only, (E) minor groove only, and (F) non-side chain-base hydrogen bonds in both major and minor grooves. *P*-values are displayed on top of the boxplots.

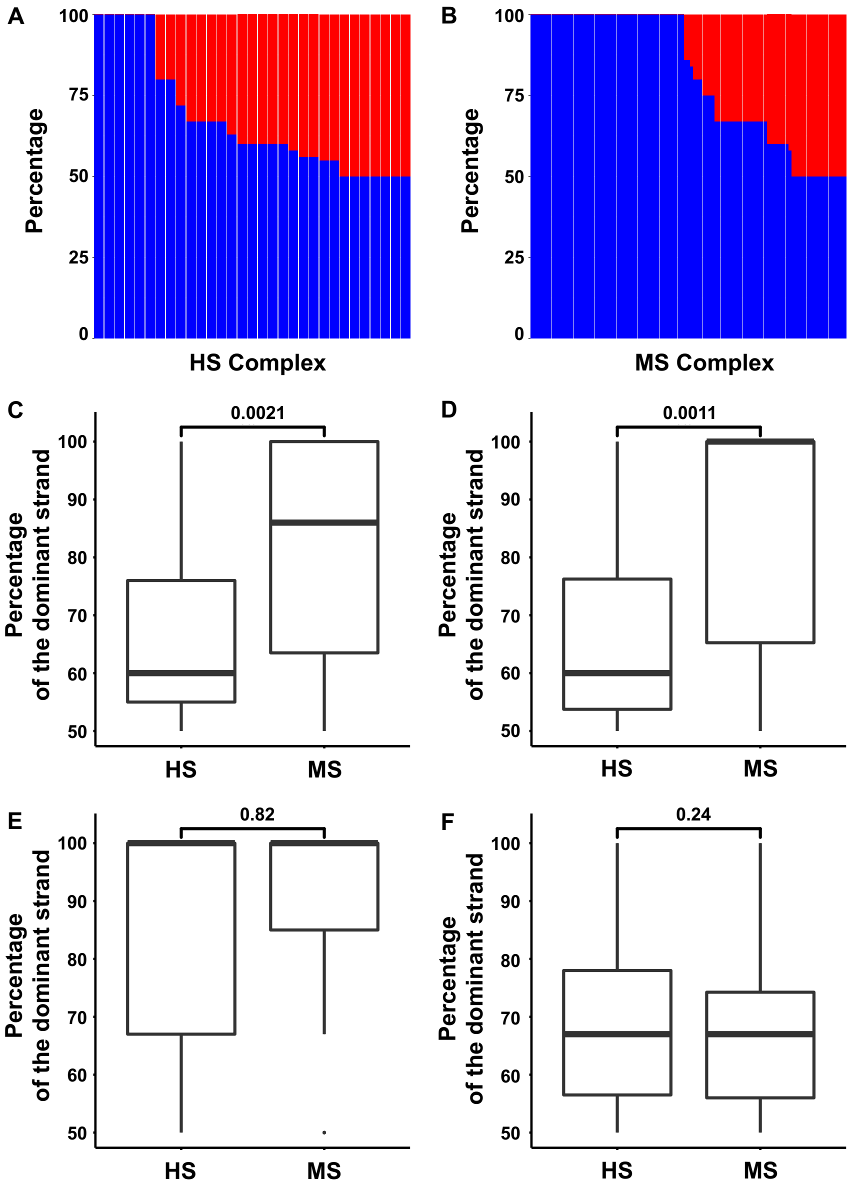

Figure S2. Comparison of side chain-base hydrogen bond energy with FIRST (-0.6 kcal/mol cutoff) of each strand of DNA between the HS and MS DNA-binding proteins for: (A) both major and minor grooves, (B) major groove, (C) minor groove, and (D) non-side chain-base hydrogen bonds in both major and minor grooves.

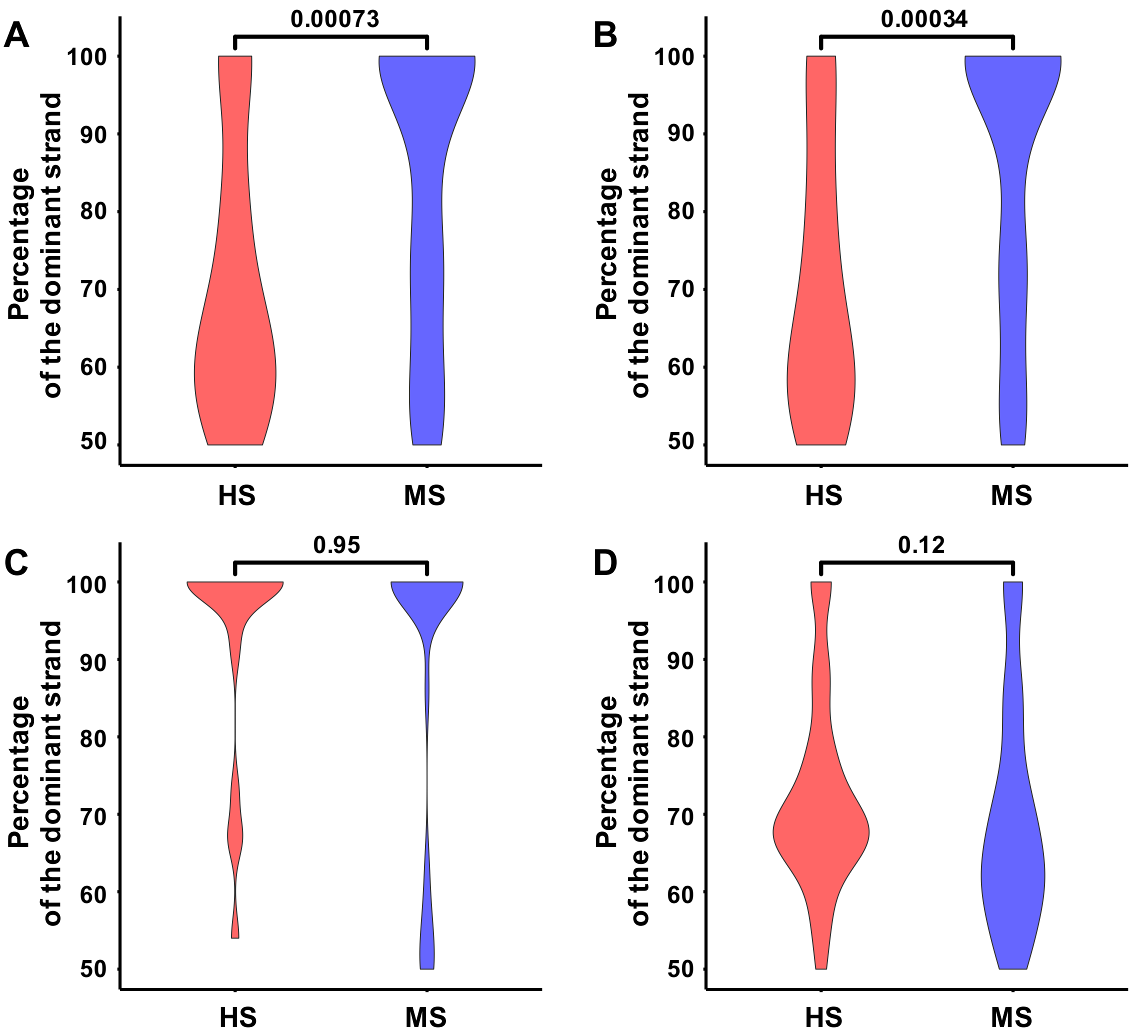

Figure S3. Comparison of chain-based and domain-based analyses of the number of side chain-base hydrogen bonds of two strands between the HS and MS groups. Both major and minor grooves were considered.

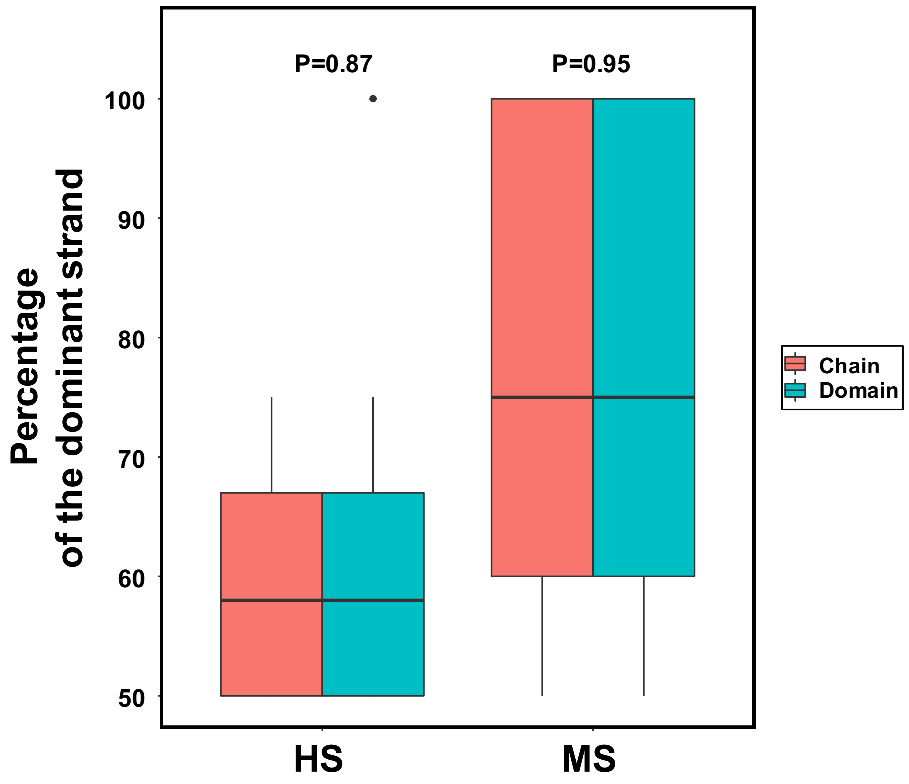

Figure S4. Comparison of the number of DNA bases involved in hydrogen bonding with side chains from FIRST (-0.6 kcal/mol cutoff) for: (A) both major and minor grooves, (B) major groove, and (C) minor groove, between HS and MS DNA-binding proteins.

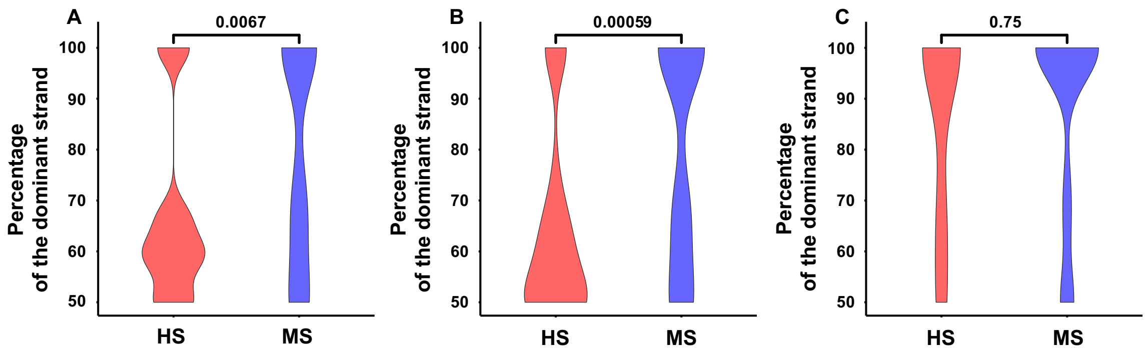

Figure S5. Base pairs involved in hydrogen bonding with residue side chains (red) in 50/50 cases in the HS group (A) and MS group (B) with HBPLUS. Individual bases that are involved in hydrogen bonding with residue side chains are shown in blue font.

A

1AZ0_BC_BD_B00
AAGATATCTT
TTCTATAGAA

1BHM_AC_AD_A00
TATGGATCCATA
ATACCTAGGTAT

1IAW_AC_AD_A02
GCCACGCCGGCGTGGC
CGGTGCGGCCGCACCG

1DC1_AC_AW_A01
ATACTCGAGTAT
TATGAGCTCATA

2FL3_AC_AD_A00
CCAGCGCTGG
GGTCGCGACC

1VRR_AC_AD_A00
TTATAGATCTATAA
AATATCTAGATATT

4ABT_AE_AH_A00
GCGCCGGCGC
CGCGGCCGCG

1YFI_BE_BF_B00
CCCCCGGGGG
GGGGGCCCCC

3FC3_BC_BD_B01
CTCGACGTA
GAGCTGCAT

2E52_DF_DH_D00
GCCAAGCTTGGC
CGGTTCGAACCG

2VLA_AL_AM_A00
GGTACCCGTGGA
CCATGGGCACCT

6EKO_AE_AF_A00
CGCTCCCGGAGCG
GCGAGGCCCTCGC

3NDH_AC_AD_A00
GTACGCGATG
CATGCGCTAC

1PVI_AC_AD_A00
GACCAGCTGGTC
CTGGTCGACCAG

1KC6_BE_BF_B00
CCGGTCGACCGG
GGCCAGCTGGCC

B

4H10_AC_AD_A00
GGAACACGTGACCC
CCTTGTGCACTGGG

3U3W_AY_AZ_A01
CTATGCAATATTTCATAT
GATACGTTATAAAGTATA

1ZS4_AT_AU_A00
ATTCGTGCAAACAAACGCAACGAGGT
TAAGCACGTTTGTTTGCGTTGCTCCA

1BL0_AB_AC_A01
GGATTTAGCAAAACGTGGCATC
CCTAAATCGTTTTGCACCGTAG

1BL0_AB_AC_A02
GGATTTAGCAAAACGTGGCATC
CCTAAATCGTTTTGCACCGTAG

1CF7_AC_AD_A00
TTTTCGCGCGGTTTT
AAAAGCGCGCCAAAA

1GD2_EA_EB_E00
GGTTACGTAACC
CCAATGCATTGG

1IC8_AE_AF_A01
CTTGGTTAATAATTCACCAGA
GAACCAATTATTAAGTGGTCT

1IC8_AE_AF_A02
CTTGGTTAATAATTCACCAGA
GAACCAATTATTAAGTGGTCT

1KB2_AC_AD_A00
CACGGTTCACGAGGTTCA
GTGCCAAGTGCTCCAAGT

1LMB_31_32_300
ATACCACTGGCGGTGATAT
TATGGTGACCGCCACTATA

1MHD_AC_AD_A00
CAGTCTAGACATA
GTCAGATCTGTAT

1ZRE_AW_AX_A02
ATTTCGAAAAATGGGAT
TAAAGCTTTTTACCCTA

2H27_AB_AC_A00
CCGGAACTTCG
GGCCTTGAAGC

2R5Y_BC_BD_B00
CTCTATGATTTATGGGCTG
GAGATACTAAATACCCGAC

2WT7_AC_AD_A00
AATTGCTGACTCATAG
TTAACGACTGAGTATC

2YVH_DE_DF_D00
TAACTGTACCGACC
ATTGACATGGCTGG

3G97_AC_AD_A00
GGAACCCAATGTTCT
CCTTGGGTTACAAGA

3PVV_BE_BF_B00
CGTTGTCCACAAC
GCAACAGGTGTTG

6ON0_AC_AN_A00
TTATAGCTAGCTATAA
AATATCGATCGATATT

3ZKC_BC_BD_B00
AAGTTCTCTTTAGAGAACAA
TTCAAGAGAAATCTCTTGTT

6CRO_AR_AU_A00
CTATCACCGCGGGTGATAC
GATAGTGGCGCCCACTATG

4ON0_BE_BF_B00
ATTAGAGAACCCTGATGTTAA
TAATCTCTTGGGACTACAATT

2XSD_CA_CB_C01
ATGCATGAGG
TACGTACTCC

2XSD_CA_CB_C02
ATGCATGAGG
TACGTACTCC

3A5T_AC_AD_A00
CTGATGAGTCAGCAC
GACTACTCAGTCGTG

3W3C_AB_AC_A00
GTGGGATTTCATGATGAAACGAG
CACCCTAAAGTACTACTTTGCTC

3H0D_BC_BD_B01
ATTAAGGTCAAATATAGTCAAAATA
TAATTCCAGTTTATATCAGTTTTAT

3W6V_AB_AC_A00
GTGAACCCGCCAAC
CACTTGGGCGGTTG

4LDX_AC_AD_B02
TTGTCTCCCTTTGGGAGACAA
AACAGAGGGAAACCCTCTGTT

Figure S6. Base pairs involved in hydrogen bonding with residue side chains (red) in 50/50 cases in the HS group (A) and MS group (B) identified by FIRST (-0.6 kcal/mol cutoff). Individual bases that are involved in hydrogen bonding with residue side chains are shown in blue font.

A

1AZ0_BC_BD_B00
AAGATATCTT
TTCTATAGAA

1BHM_AC_AD_A00
TATGGATCCATA
ATACCTAGGTAT

1DC1_AC_AW_A01
ATACTCGAGTAT
TATGAGCTCATA

2FL3_AC_AD_A00
CCAGCGCTGG
GGTCGCGACC

3DVO_DG_DH_D00
GAGTCCACCGGTGGACTC
CTCAGGTGGCCACCTGAG

4ABT_AE_AH_A00
GCGCCGGCGC
CGCGGCCGCG

1YFI_BE_BF_B00
CCCCCGGGGG
GGGGGCCCCC

2VLA_AL_AM_A00
GGTACCCGTGGA
CCATGGGCACCT

1PVI_AC_AD_A00
GACCAGCTGGTC
CTGGTCGACCAG

1KC6_BE_BF_B00
CCGGTCGACCGG
GGCCAGCTGGCC

2E52_DF_DH_D00
GCCAAGCTTGGC
CGGTTCGAACCG

3NDH_AC_AD_A00
GTACGCGATG
CATGCGCTAC

1ERI_AB_AC_A00
CGCGAATTCGCG
GCGCTTAAGCGC

B

3COA_CA_CB_C00
TGGTTTGTTTTGCTTG
ACCAAACAAAACGAAC

4IX7_AC_AD_A00
TTCCAATTGGAA
AAGGTTAACCTT

3U3W_AY_AZ_A01
CTATGCAATATTTCATAT
GATACGTTATAAAGTATA

1ZS4_AT_AU_A00
ATTCGTGCAAACAAACGCAACGAGGT
TAAGCACGTTTGTTTGCGTTGCTCCA

1CF7_AC_AD_A00
TTTTCGCGCGGTTTT
AAAAGCGCGCCAAAA

1IC8_AE_AF_A02
CTTGGTTAATAATTCACCAGA
GAACCAATTATTAAGTGGTCT

1LMB_31_32_300
ATACCACTGGCGGTGATAT
TATGGTGACCGCCACTATA

1NKP_DH_DJ_D00
GAGTAGCACGTGCTACTC
CTCATCGTGCACGATGAG

2WT7_AC_AD_A00
AATTGCTGACTCATAG
TTAACGACTGAGTATC

3W6V_AB_AC_A00
GTGAACCCGCCAAC
CACTTGGGCGGTTG

3G97_AC_AD_A00
GGAACCCAATGTTCT
CCTTGGGTTACAAGA

3PVV_BE_BF_B00
CGTTGTCCACAAC
GCAACAGGTGTTG

6ON0_AC_AN_A00
TTATAGCTAGCTATAA
AATATCGATCGATATT

3S8Q_AC_AD_A00
TGTGACTTATAGTCCGTG
ACACTGAATATCAGGCAC

3ZKC_BC_BD_B00
AAGTTCTCTTTAGAGAACAA
TTCAAGAGAAATCTCTTGTT

6CRO_AR_AU_A00
CTATCACCGCGGGTGATAC
GATAGTGGCGCCCACTATG

2XSD_CA_CB_C01
ATGCATGAGG
TACGTACTCC

2XSD_CA_CB_C02
ATGCATGAGG
TACGTACTCC

3W3C_AB_AC_A00
GTGGGATTTCATGATGAAACGAG
CACCCTAAAGTACTACTTTGCTC

4HF1_AC_AD_A00
ATAAATCCACACAGTTTGTATTGTTTTGT
TATTTAGGTGTGTCAAACATAACAAAACA

1RIO_HT_HU_H00
CCATGTCAAGCACTGGCGGTGATACCG
GGTACAGTTCGTGACCGCCACTATGGC

4MTE_BY_BZ_B01
GAAGTGTGATATTATAACATTTCATGACTA
CTTCACACTATAATATTGTAAAGTACTGAT

2H7H_AX_AY_A00
CGTCGATGACTCATCGACG
GCAGCTACTGAGTAGCTGC

2P5L_CA_CB_C00
CATGAATAAAAATTCAAG
GTACTTATTTTTAAGTTC

Figure S7. Comparison of side chain-base hydrogen-bonding base pairs with FIRST (-0.6 kcal/mol cutoff) between HS and MS groups in (A) both major and minor grooves and (B) major groove.

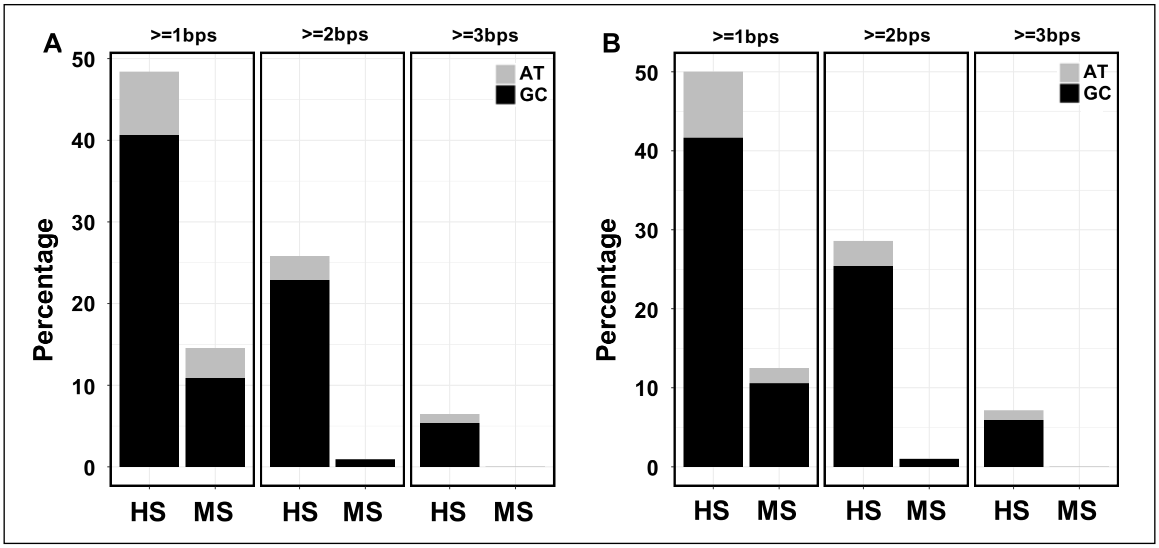

Figure S8. Secondary structure preferences of highly specific DNA-binding protein and multi-specific DNA-binding protein. (A) Highly specific DNA-binding protein representative (PDBID: 1VRR; protein chain: A; DNA chains: C and D). Strand and coil secondary structure types (magenta) are involved in side chain-base hydrogen bonds (blue dash line). Two DNA bases involved in hydrogen bonds with protein side chains, A5 and T10, are paired bases; hydrogen bonds between this pair are shown in red dash line. (B) Multi-specific DNA-binding protein representative (PDBID: 1IC8; protein chain: A; DNA chains: E and F). Residues involved in hydrogen bonding are in helical conformation (magenta).

A

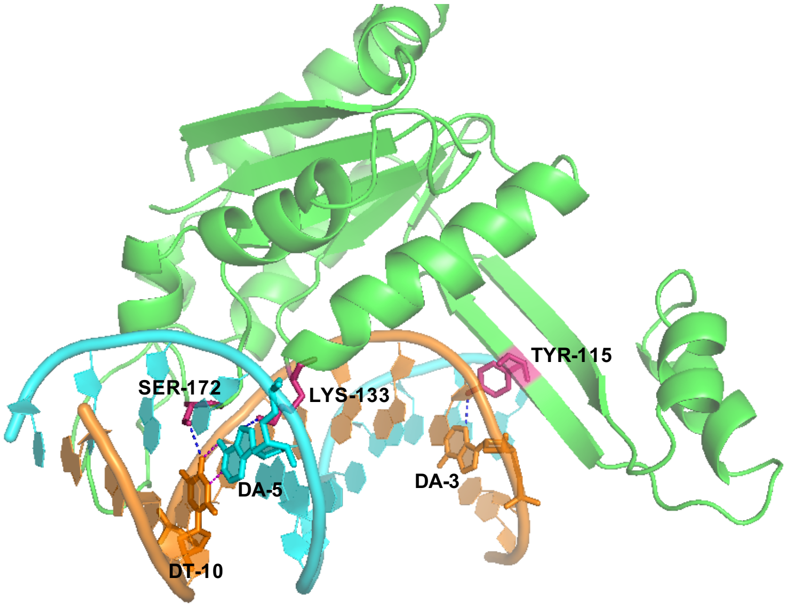

B

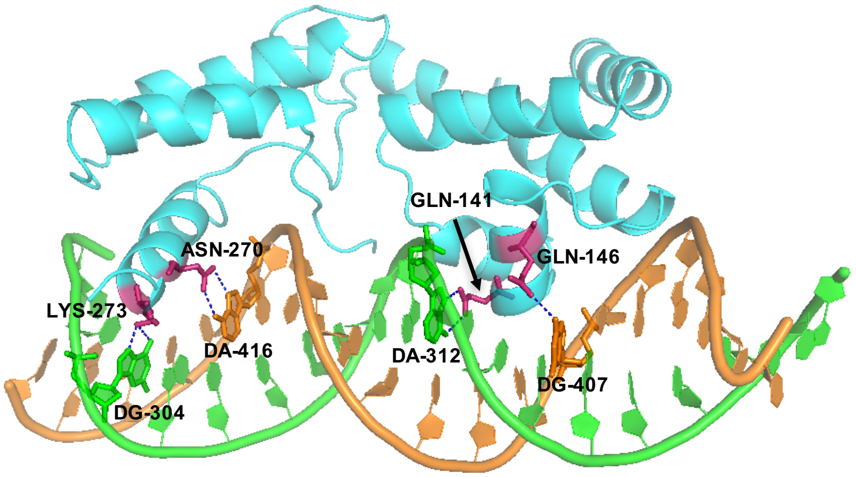

Figure S9. Propensities of secondary structure types of residues involved in side chain-base hydrogen bonds with FIRST (-0.6 kcal/mol cutoff). (A, C) both major and minor grooves and (B, D) major groove only. Propensities are calculated over the relative frequencies of secondary structure types of base-contacting residues (A, B) and all DNA hydrogen-bonding residues (C, D).

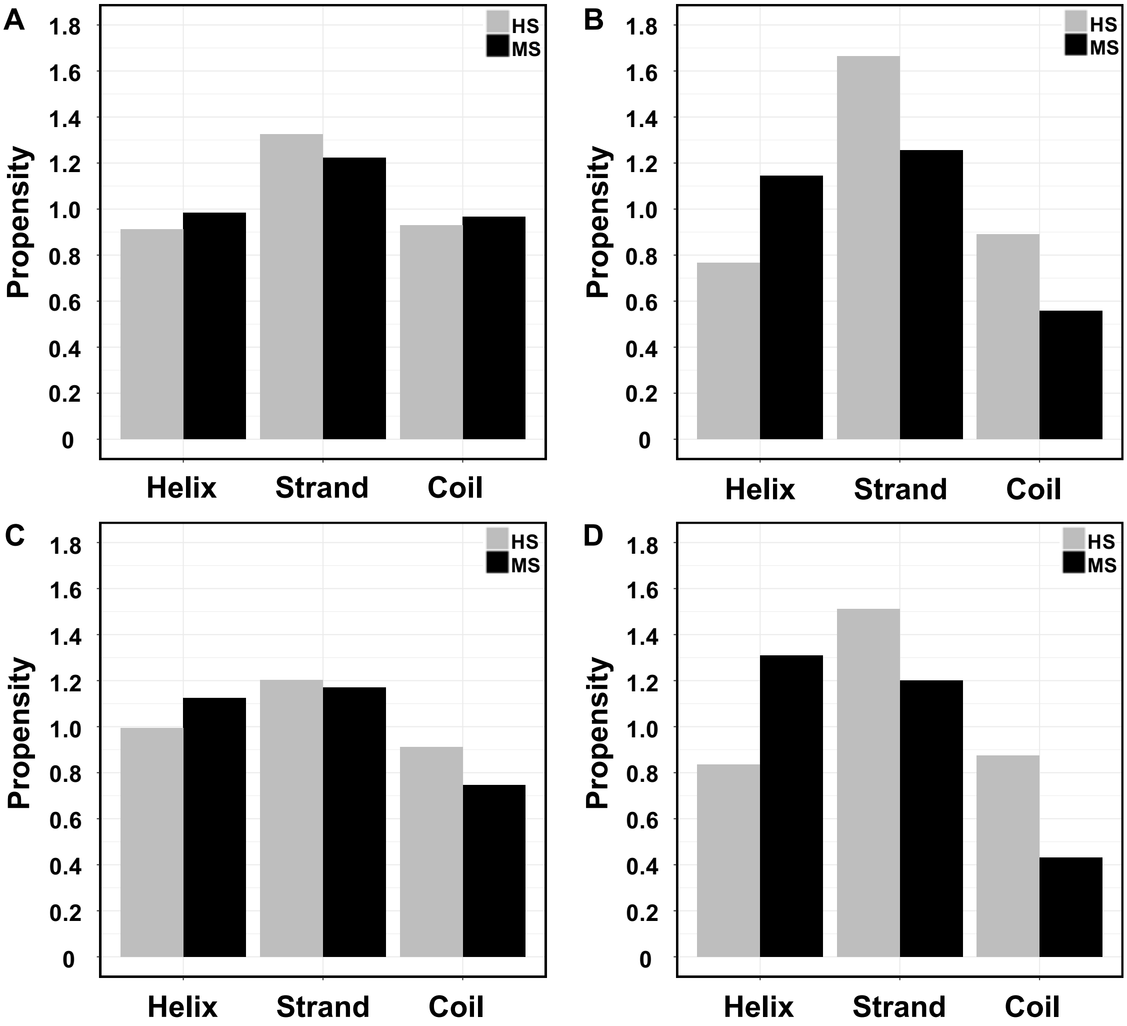

Supplement: gkz963_Supplemental_File [file gkz963_supplemental_file.pdf]
